# Transcriptionally distinct B cell profiles in systemic immune tissues and peritoneal cavity of Atlantic salmon (*Salmo salar*) infected with salmonid alphavirus subtype 3

**DOI:** 10.3389/fimmu.2024.1504836

**Published:** 2024-12-03

**Authors:** Shiferaw Jenberie, Simen Rød Sandve, Thu-Hien To, Matthew Peter Kent, Espen Rimstad, Jorunn B. Jørgensen, Ingvill Jensen

**Affiliations:** ^1^ Norwegian College of Fishery Science, Faculty of Biosciences, Fisheries & Economics, UiT- the Arctic University of Norway, Tromsø, Norway; ^2^ Center for Integrative Genetics (CIGENE), Department of Animal and Aquacultural Sciences, Faculty of Biosciences, Norwegian University of Life Sciences, Ås, Norway; ^3^ Faculty of Veterinary Medicine, Norwegian University of Life Sciences, Ås, Norway

**Keywords:** B cells, transcriptome, Atlantic salmon, peritoneal cavity, salmonid alphavirus, teleost, *Salmo salar*

## Abstract

Teleost B cells producing neutralizing antibodies contribute to protection against salmonid alphavirus (SAV) infection, the etiological agent of pancreas disease, thereby reducing mortality and disease severity. Our previous studies show differences in B cell responses between the systemic immune tissues (head kidney (HK) and spleen) and the peritoneal cavity (PerC) after intraperitoneal SAV3 infection in Atlantic salmon (*Salmo salar*) where the response in PerC dominates at the late time points. By employing the same infection model, we aimed to further characterize these B cells. Immunophenotyping of teleost B cells is challenging due to limited availability of markers; however, RNA-seq opens an opportunity to explore differences in transcriptomic responses of these cells. Our analysis identified 334, 259 and 613 differentially expressed genes (DEGs) in Atlantic salmon IgM^+^IgD^+^ B cells from HK, spleen, and PerC, respectively, at 6 weeks post SAV3 infection. Of these, only 34 were common to all the three immune sites. Additionally, out of the top 100 genes with the highest fold change in expression, only four genes were common across B cells from the three sites. Functional enrichment analyses of DEGs using KEGG and GO databases demonstrated differences in enriched innate immune signaling and the cytokine-cytokine interaction pathways in B cells across the sites, with varying numbers of genes involved. Overall, these findings show the presence of transcriptionally distinct B cell subsets with innate immune functions in HK, spleen and PerC of SAV3-infected Atlantic salmon. Further, our data provide new insights into the immunoregulatory role of fish B cells through the differential expression of various cytokine ligands and receptors and will be a useful resource for further studies into B cell immune compartments.

## 1 Introduction

Teleost B cells have important roles in both innate and adaptive immunity and are of interest due to the evolutionary position of this diverse group of animals (1–3). In aquaculture, disease prophylaxis relies on protective antibody responses induced by vaccination, emphasizing the need for a comprehensive understanding of fish B cells (4, 5). While three immunoglobulin (Ig) isotypes have been identified in teleost fish: IgM, IgD and IgT, humoral immunity relies on unswitched IgM during both primary and secondary responses (6, 7). IgM is the principal systemic Ig abundant in serum (6), while new studies suggest that IgD contributes to both systemic and mucosal responses (8, 9). IgT serves as the predominant Ig at mucosal surfaces and is secreted by a separate B cell lineage (10–13). While lymph nodes and bone marrow are lacking, the major systemic lymphoid tissues in teleosts involved in B cell generation and activation are the anterior kidney (head kidney-HK) and spleen (14). The majority of naïve teleost B cells co-express IgM and IgD (reviewed in (15)). Upon activation, naive B cells can downregulate surface IgM/IgD expression, leading to differentiation into antibody secreting cells with characteristics of plasmablasts/plasma cells (16).

A functional similarity has been proposed between fish B cells and the innate-like B1 cells of higher vertebrates. This is based on an antibody repertoire with restricted complexity/specificity, surface CD5 expression, high intracellular complexity, high surface IgM but low IgD expression, extended survival *in vitro* and weak proliferation after B cell receptor (BCR) engagement (2, 7). Also, high phagocytic capacity and responsiveness to innate stimuli through toll like receptors (TLRs) are shared characteristics of teleost B cells and mammalian B1 B cells (2, 17, 18). Further knowledge about teleost B cell subsets has been scarce, however, recent studies have identified transcriptionally distinct B cell clusters within and between different tissues in rainbow trout and Atlantic salmon (19–22).

In aquaculture, intraperitoneal (ip) injection is the primary method for administering vaccines, highlighting the need to understand the local B cell response in the peritoneal cavity (PerC). Research has shown that PerC adipose tissue of teleosts can function as a secondary immune site, affecting the immune response to antigens delivered by ip injection (23–25). This could be due to its ability to provide a favorable milieu for the activation, differentiation, and long-term survival of antibody-secreting cells, along with retaining antigens for an extended period (16, 23, 26–28). In Atlantic salmon infected with salmonid alphavirus (SAV3) or *Piscirickettsia salmonis* by ip injection, we found higher frequencies and the most prolonged presence of both total and antigen specific IgM secreting cells in the PerC compared to HK and spleen (26–28). These findings prompted further investigations into the characteristics of local versus systemic B cell responses in Atlantic salmon. In the current study, using the same infection model as previously, i.e., ip infection with SAV3 (27, 28), transcriptomic responses of sorted IgM^+^IgD^+^ B cells from PerC, HK and spleen were analyzed. The results revealed concurrent virus-induced enrichment of various innate immune signaling pathways and the cytokine-cytokine interaction pathway in B cells from all examined sites underscoring their innate and immunoregulatory potential, possibly shaping the overall immune response to infection. These findings demonstrate the presence of transcriptionally distinct B cell subsets within the immune sites of Atlantic salmon following SAV3 infection, while also supporting the current understanding of the innate immune functions of teleost B cells.

## 2 Materials and methods

### 2.1 Fish and SAV3 challenge

An infection experiment was conducted at the Tromsø Aquaculture Research station, Tromsø, Norway. The fish were non-vaccinated Atlantic salmon (*Salmo salar*) reared at the research station and found free of SAV, infectious salmon anemia virus (HPR0 and HPRdel), piscine orthoreovirus 1, salmon gill poxvirus, piscine myocarditis virus and infectious pancreatic necrosis virus by RT-qPCR (Pharmaq Analytic). The fish weighed around 220g at the start of the experiment and were kept in running seawater at 12°C, exposed to 24 hours light, and fed with commercial dry feed. Prior to any handling, they were starved for 12 hours. The fish were randomly selected, anesthetized by bath immersion in benzocaine chloride (0.5 g/10 L) and injected ip with 0.15 mL (10^2^ TCID_50_) of the virus clone rSAV3 (29) or with 0.15 mL PBS (control), after which they were kept in separate tanks. There were no mortalities in the experimental groups. The experiment was approved by the Norwegian Animal Research Authority (NFDA; ID: 27026), according to the European Union Directive 2010/63/EU and was performed in accordance with the guidelines outlined in the current animal welfare regulations of Norway (FOR-1996-01-15-23).

### 2.2 Sampling and leukocyte isolation

HK and spleen, along with PerC washes, were obtained from six control and six SAV3-infected fish at 3- and 6-weeks post injection (wpi) as previously described (27, 30). The PerC cells were collected with three successive washes, each with 1 mL PBS containing 2% fetal bovine serum (FBS) and 20 U/mL heparin, through an incision made along the midventral line while positioning the fish on its back. The cell suspension was then mixed with 2 mL ice-cold transport medium (L-15 with 100 U/mL penicillin, 0.1 mg/mL streptomycin, 2% FBS and 20 U/mL heparin). HK and spleen tissues were passed through 100 µm cell strainers (Falcon). Cell suspensions from the three sites were layered onto discontinuous 25/54% Percoll gradients. Following centrifugation at 400 x g 4 °C for 40 minutes, cells were collected from the interface of the gradients, washed once with L-15, counted (Countess II FL; Invitrogen), resuspended in PBS containing 1% FBS, and immediately stained for FACS analysis.

### 2.3 FACS of B cells

For the HK and spleen, up to 1.5x10^7^ total leukocytes were transferred into 1.5 mL tubes, while 96-well U-bottomed plates were used for PerC leukocytes that rarely reached a total count of 1.5x10^6^. The cells were spun down and washed once by resuspending in FACS buffer (FB, Dulbecco’s PBS with 1% BSA). Live cells were stained with Alexa Fluor 647 conjugated anti-trout IgM mAb (IgF1-18 (6-1-18); 2 µg/mL; in-house conjugated using ThermoFisher’s Alexa Fluor 647 labeling kit for detection in the APC channel) (31) and biotinylated anti-trout IgD mAb (2 µg/mL; in-house biotinylated using ThermoFisher’s EZ-Link™ NHS-PEG biotinylation kit). The anti-trout IgD antibody is described in (32). A peptide sequence identical to the trout peptide used to generate the antibody is present in the Atlantic salmon IGH constant δ2 domain (33). After two washes, cells were incubated with PE-streptavidin (1 µg/mL; BioLegend) and viability dye V450 (2ul/mL; ThermoFisher, excited by the violet laser with emission in the blue spectrum). Cells were kept on ice and all incubations were carried out at 4 °C for 20 minutes protected from light. The washing and staining steps were done using FB and performed by spinning at 500 x g 4 °C for 5 minutes. Double-positive (IgM^+^IgD^+^) and single positive (IgM^+^IgD^-^) B cells were FACS sorted on a BD FACS Aria III (BD Biosciences) directly into tubes with RNA lysis buffer (RLT buffer, Qiagen). The IgM^-^IgD^+^ cells were not sorted because of their low frequency (< 2%), which made it difficult to gate on the population of interest with high confidence (**Supplementary Figure 1**). Unstained and single-stained cells were used to adjust voltages for the various parameters. Doublets (FSC-A vs FSC-H) and dead cells (V450^+^) were excluded before gating on the lymphocyte population (**Supplementary Figure 1**). When available, up to 200,000 sorted cells were collected from each sample. Sorted cell lysates were stored at -80 °C. The purity of sorted cells was checked by flow cytometry using aliquots of cells sorted directly into the transport medium. The purity of the sorted IgM^+^IgD^+^ B cells, the most dominant B cell subset, was > 98% for all the samples employed in this study (**Supplementary Figure 1**). The FACS data on B cell frequency, mean fluorescence intensity (MFI) and size were statistical analyzed using the Mann-Whitney U test to compare the control and infected groups, with a p-value threshold set at 0.05.

### 2.4 RNA extraction

After thawing, the cell lysate volumes were adjusted by adding an equal volume of the lysis buffer to increase efficiency of RNA isolation (34). Total RNA was extracted using the RNeasy Micro kit following the manufacturer’s protocol (Qiagen). The quantity of RNA was measured on Qubit 4 using the high sensitivity (HS) RNA kit (ThermoFisher). RNA integrity (RIN) was determined on an Agilent 2100 Bioanalyzer using Agilent RNA 6000 Pico kit. All samples used herein had RIN values above 8. RNA samples were stored at -80°C.

### 2.5 Library preparation and sequencing

Due to low RNA yield for some of the samples, an ultra-low input library preparation kit was used for all samples. SMART-Seq v4 (SSv4) technology, which uses oligo (dT) priming, was used to obtain full-length transcripts (TaKaRa Bio). First-strand cDNA synthesis, cDNA amplification by LD-PCR, purification of amplified cDNA, and determination of cDNA quality and quantity were performed according to the manufacturer’s protocol. Library preparation and library sequencing were performed at Novogene, Cambridge, UK, employing the NovaSeq PE150 sequencing platform. A total of 57 double (IgM^+^IgD^+^) and 24 single (IgM^+^IgD^-^) positive samples were sequenced. The sequencing reaction generated more than six G raw data per sample with Q30 ≥ 85%. Raw reads were deposited in NCBI’s Sequence Read Archive (SRA) under Accession No. SUB14129322.

### 2.6 Read quality pre-processing and mapping

The quality of raw reads was assessed using FastQC v.0.11.9, and quality filtering and pre-processing were performed using fastp v.0.23.2 (35). A decoy-aware transcriptome index was prepared from NCBI’s most recent Atlantic salmon genome assembly files (GCF_905237065.1_Ssal_v3.1_genomic.fna) and gene model annotation (GCF_905237065.1_Ssal_v3.1_genomic.gff) using the software Salmon v.1.4.0 with the keepDuplicates option passed (36). Mapping of cleaned reads to the transcriptome index and quantification of reads were performed using the Salmon quant command. Salmon counts data were imported for further analyses using a Bioconductor package tximport v.1.26.1 (37). The immunoglobulin µ (IgM) genes were not present in the Atlantic salmon gene model annotation file (GCF_905237065.1_Ssal_v3.1_genomic.gff), and hence, we manually annotated them using available IgM sequences on the NCBI’s website (**Supplementary Table 1**). This manual annotation file was used to quantify the IgM transcripts. A box plot and principal component analysis (PCA) were employed to visually evaluate and compare the distribution of regularized log2 (rlog) transformed raw read data within and across samples. Outlier samples exhibiting a distinct data distribution or not clustered within their respective groups, were excluded from the analyses.

### 2.7 Differential expression and pathway enrichment analyses

Differential expression and pathway enrichment analyses were carried out utilizing R scripts (R v.4.2.2) and Bioconductor packages. The gene-level count data were obtained using Salmon (36) and differential expression of genes was analyzed using DESeq2 v.1.36.0 (38). Lowly expressed genes (≤ 10 counts in total across all fish) were discarded before analyses. The Benjamini-Hochberg false discovery rate for multiple testing correction was applied, and genes with adjusted p-value (padj) < 0.05 and absolute value log2 fold change (LFC) > 1 were considered differentially expressed. Heat map visualizations were carried out using the R package pheatmap(). The Kyoto Encyclopedia of Genes and Genomes (KEGG) and gene ontology (GO) databases were utilized to identify enriched biological pathways and overrepresented GO terms, respectively, using ClusterProfiler with the ‘universe’ option set to all expressed genes (39). Note that a substantial proportion of differentially expressed genes (DEGs) from the three sites were not annotated by either KEGG or GO databases, owing to the quality of the Atlantic salmon genome annotation. In addition, the IgM genes, which were annotated manually in this study, are not present in the databases employed for the enrichment analyses. Consequently, functional enrichment for these genes was not feasible. Pathways or GO terms with a Benjamini-Hochberg corrected p-value (padj) < 0.05 were considered significantly enriched

## 3 Results

### 3.1 Phenotypic characteristics of sorted B cells

**Figure 1:**
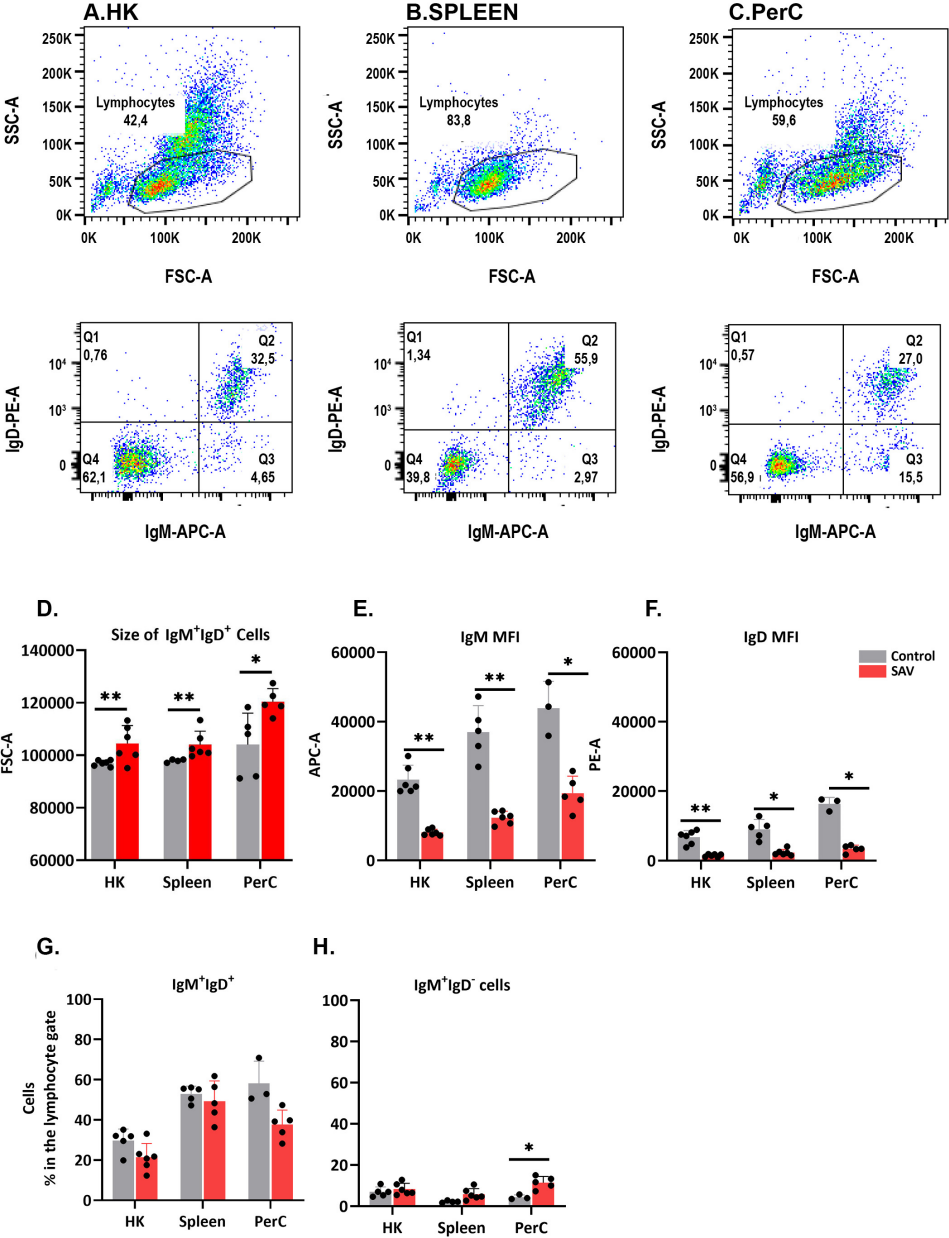
FACS analysis of B cells isolated from HK, spleen, and PerC. Dot plot showing the FSC vs. SSC characteristics of leukocytes from HK **(A)**, spleen **(B)** and PerC **(C)**. **(D)** Median size (FSC-A), **(E)** IgM median fluorescence intensity (MFI) and **(F)** IgD MFI of IgM^+^IgD^+^ B cells in control or SAV infected Atlantic salmon at 6 wpi. **(G)** Frequency of IgM^+^IgD^+^ and **(H)** IgM^+^IgD^-^ B cells in control or SAV infected Atlantic salmon at 6 wpi (n = 3 to 6).

The phenotypic characteristics of B cells from HK, spleen and PerC of control and SAV3-infected fish were studied using FACS (**Figure 1**). At 6 wpi, SAV-infected fish showed a significant increase in the size of IgM^+^IgD^+^ cells, along with a decrease in surface expression of IgM and IgD, compared to control fish across the three sites (**Figure 1**). This suggests the emergence of a virus-induced B cell phenotype resembling plasmablasts or plasma cells. While not statistically significant, there was a general trend of a decline in the frequency of IgM^+^IgD^+^ cells and an increase in the frequency of IgM^+^IgD^-^ cells across the three sites of infected fish at 6 wpi (**Figure 1**). Flow data at 3 wpi were recorded from a limited number of individuals and were excluded from the analysis.

### 3.2 Quality control of raw reads and exploratory data analyses

RNA-seq data of sorted B cells used in this study demonstrated raw read quality scores > 87% (Q30). However, samples exhibiting low mapping rates (< 42%) after quality filtering and trimming were excluded from further analysis. These included 12 PerC, 2 HK and 2 spleen IgM^+^IgD^+^ B cell samples at 3 wpi, and 2 PerC and 3 spleen IgM^+^IgD^+^ B cell samples at 6 wpi (**Supplementary Figure 2**). IgM^+^IgD^-^ cells were discarded because of low RNA-seq data quality, likely caused by RNA quantities in the lower limit of the library preparation kit. The average mapping rate of samples included in this study was 85%, equivalent to an average of 20.8 million reads per sample (**Supplementary Figure 2**). PCA showed a distinct separation between the control and infected groups across the three sites, albeit exhibiting considerable within-group individual variations, which is expected for outbred species such as the Atlantic salmon (**Supplementary Figure 3**). To further evaluate the purity of our sorted cells, we assessed the presence of *IgT*, *CD4* and *CD8* transcripts. *IgT* and *CD4* were not detected, while *CD8a* was found, but not differentially expressed. The detection of *CD8a* transcripts may be due to T cell contamination of the sorted B cells. However, given that mammalian B cells can express CD8 under certain pathological conditions (40), additional investigation is required to rule out the possibility of CD8 expression by Atlantic salmon B cells.

### 3.3 Transcriptionally distinct B cells across immune sites after SAV3 infection

**Figure 2:**
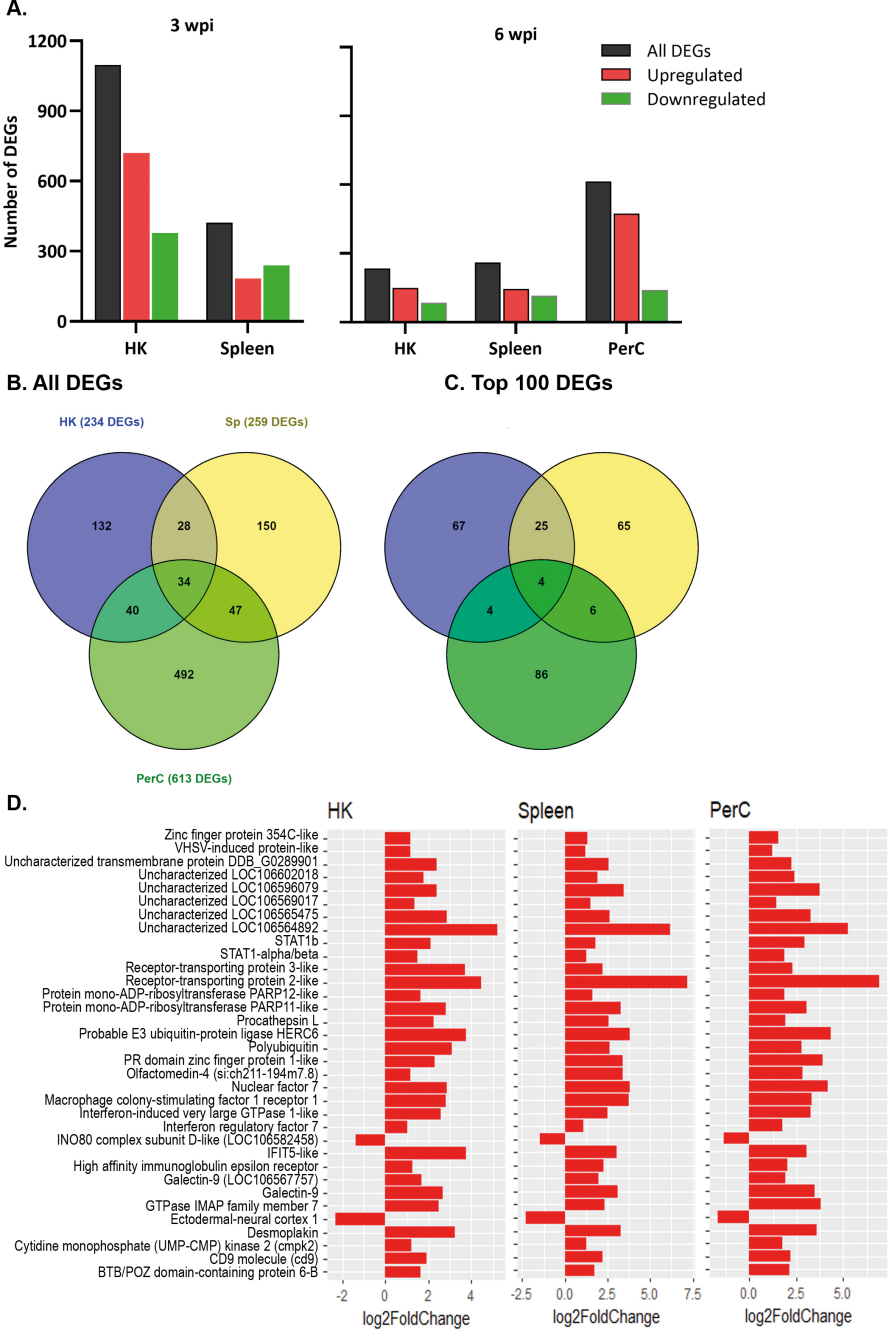
The number of differentially expressed genes (DEGs) in HK, spleen, and PerC IgM^+^IgD^+^ B cells of Atlantic salmon infected with SAV, compared to control fish. **(A)** Number of significantly upregulated or downregulated genes at 3 or 6 wpi in IgM^+^IgD^+^ B cells across the studied sites. **(B)** A Venn diagram depicting the total number of DEGs and DEGs shared between HK, spleen, and PerC B cells at 6 wpi; **(C)** the number of overlapping DEGs among the top 100 DEGs in B cells from the three sites ranked by LFC at 6 wpi. **(D)** The 34 DEGs shared among HK, spleen, and PerC B cells at 6 wpi.

**Figure 3:**
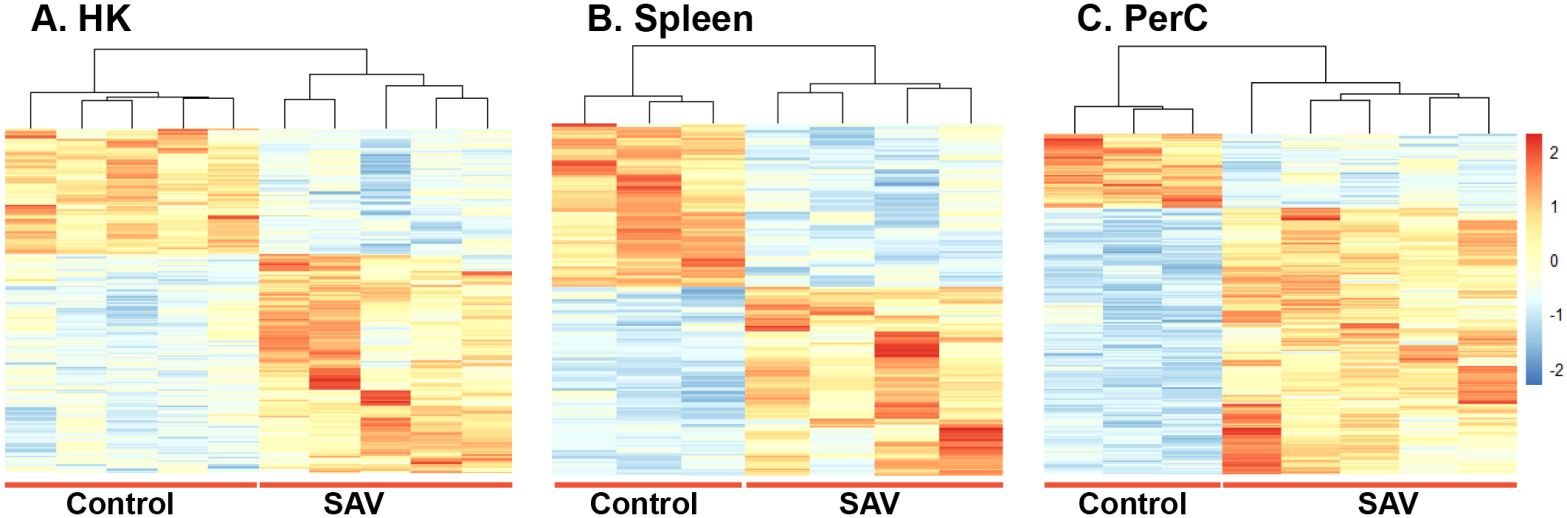
Hierarchical clustering and heat map of DEGs in **(A)** HK, **(B)** Spleen and **(C)** PerC B cells from control or SAV-infected Atlantic salmon at 6 wpi. The sidebar ranging from 2 to -2 indicates Z-score, where the red color represents upregulated genes, while the blue color represents downregulated genes. The intensity of the color corresponds to the degree of regulation, with darker shades indicating stronger regulations.

DEGs were obtained by comparing the gene expression of B cells in the SAV3-infected fish with that of the control fish. At 3 wpi, a higher number of DEGs was found in HK (1097) than in spleen (421) B cells of the infected fish (|LFC| ≥ 1 and padj < 0.05) (**Figure 2A**). At 6 wpi, HK and spleen B cells from infected fish had comparable numbers of DEGs, with 234 and 259 DEGs, respectively, while the number of DEGs in PerC B cells was ≥ 2.4-fold higher (613) (**Figure 2A**). A complete list of DEGs from the three sites and both time points can be found in **Supplementary Tables 2**-**6**. To assess the level of ‘shared’ transcriptome responses upon SAV3-infection, we then compared the lists of DEGs among B cells from the three sites. At 3 wpi, 111 DEGs were shared between HK (~10%) and spleen (~26%) B cells, while 34 DEGs were shared between HK (~15%), spleen (13%) and PerC (~6%) B cells at 6 wpi (**Figure 2B**, **Supplementary Figure 4**). **Figure 2D** illustrates the 34 shared DEGs along with their corresponding LFC values. The B cell differentiation marker gene, PR domain zinc figure protein 1-like, was one of the genes common to all the three sites. Among the top 100 DEGs with the highest LFC values, 17 were common at 3 wpi between HK and spleen B cells and 4 (receptor-transporting protein 2-like, probable E3 ubiquitin-protein ligase HERC6, nuclear factor 7, and LOC106564892) were common at 6 wpi among HK, spleen, and PerC (**Figure 2C**, **Supplementary Figure 4**). Additionally, comparing only two sites at 6 wpi, 29 of the top 100 DEGs were shared between HK and spleen, whereas PerC had only 8 and 10 of its top 100 DEGs shared with HK and spleen, respectively (**Figure 2C**). The number of DEGs and shared DEGs between HK and spleen B cells decreased from 3 to 6 wpi. Considering the direction of transcriptional responses, more of the DEGs were upregulated than downregulated, except for spleen B cells at 3 wpi (**Figure 2A**). Hierarchical clustering combined with heat maps showed consistent patterns of between-group differences in the global DEGs (**Figure 3**, **Supplementary Figure 5**), in the 25 most significantly affected DEGs (**Supplementary Figure 6**) as well as substantial individual variations within the groups.

### 3.4 PerC B cells exhibit a comparable expression of IGH genes from the two loci

**Figure 4:**
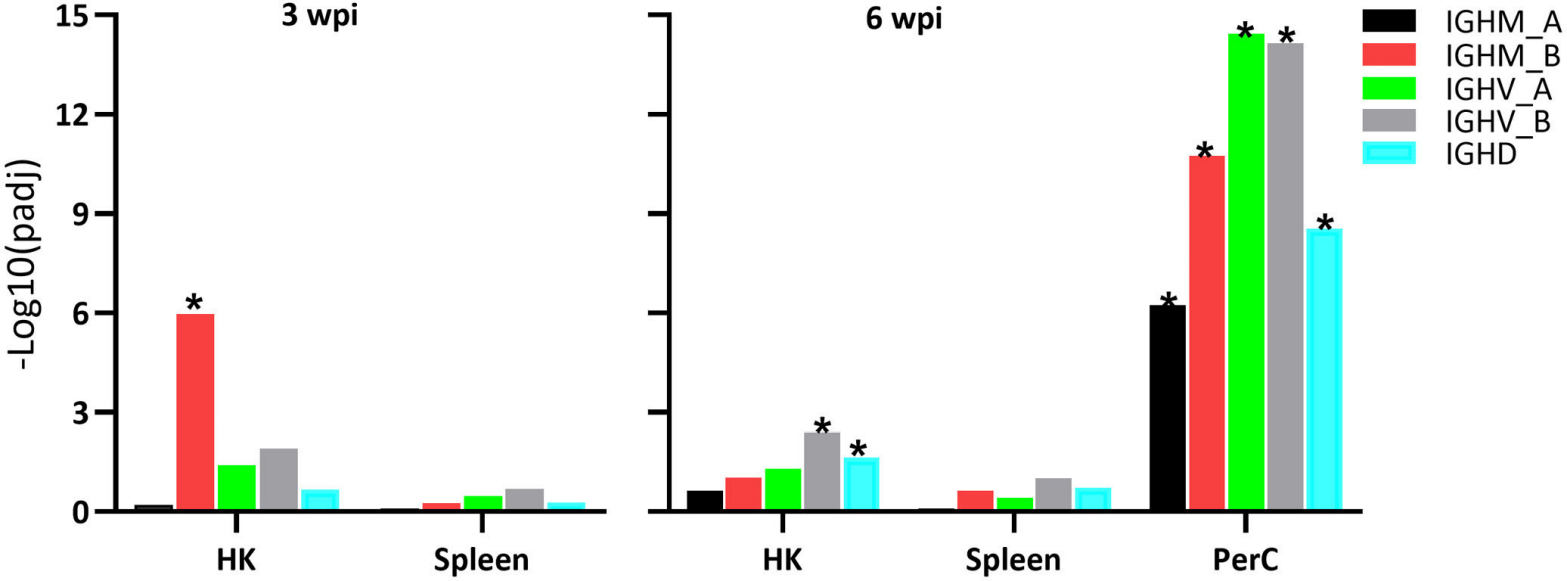
Differential expression of immunoglobulin (Ig) genes in sorted IgM^+^IgD^+^ B cells from the three sites of Atlantic salmon infected with SAV, compared to controls, at 3 and 6 wpi. IGHM_A: Ig heavy chain mu locus A; IGHM_B: Ig heavy chain mu locus B; IGHV_A: Ig heavy chain variable region locus A; IGHV_B: Ig heavy chain variable region locus B; IGHD: Ig heavy chain delta.

The two IGH loci (locus A on chromosome 6 and locus B on chromosome 3) are responsible for the transcription of Atlantic salmon IGH genes (41). Hence, both loci were manually annotated and included into our analysis, showing significant transcriptional responses in samples from SAV3-infected Atlantic salmon compared to controls (**Figure 4**). At 3 wpi, HK B cells demonstrated a significant increase in transcription of the *IGHM_B* gene, with a LFC of 1.2. At 6 wpi, PerC B cells showed a significant increase in transcription of the IGHM gene from both the A and B loci with LFC values of 4.5 (*IGHM_A*), 4.4 (*IGHM_B*), 3.4 (*IGHV_A*), 2.7 (*IGHV_B*), while in HK B cells only the I*GHV_B* gene displayed increased transcription with an LFC of 1.5. Increased transcription of *IGHD* was also found in the PerC and HK B cells at 6 wpi, with LFC of 1.2 and 2.4, respectively. In the spleen, there were no significant changes in *IGHM* or *IGHD* transcripts at both time points.

### 3.5 Innate immune signaling and cell division pathways dominated the enrichment analyses

**Figure 5:**
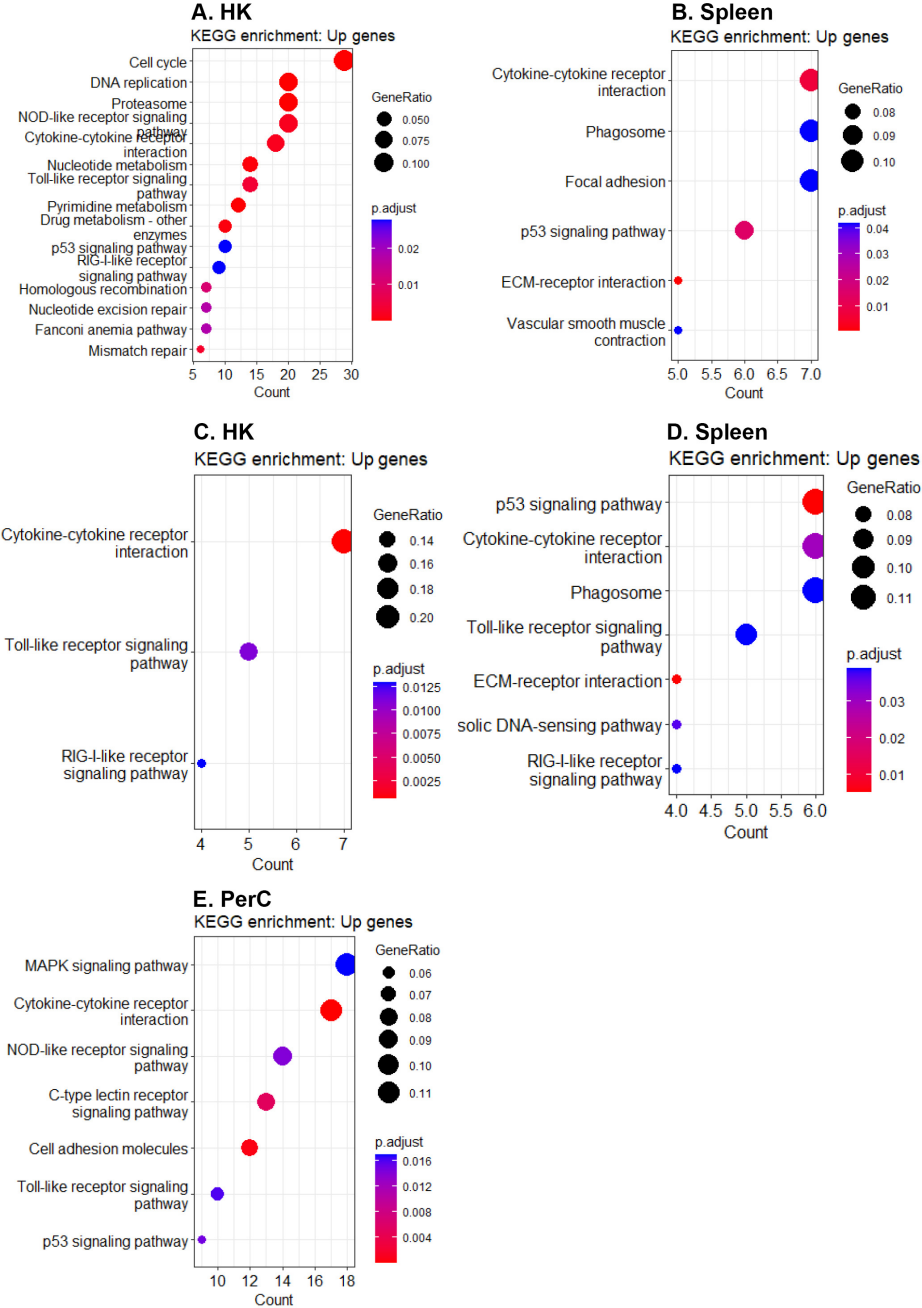
Significantly enriched KEGG pathways (padj < 0.05). Enriched KEGG pathways for upregulated genes in **(A)** HK and **(B)** spleen B cells at 3 wpi, as well as in **(C)** HK, **(D)** spleen, and **(E)** PerC B cells at 6 wpi.

At 3 wpi, 32.7% and 34.2% of the DEGs in HK and spleen B cells, respectively, were annotated into at least one KEGG pathway, while 49.0% and 46.6% of the DEGs in HK and spleen B cells, respectively, were associated with at least one over-represented GO term. Similarly, at 6 wpi, 29.5%, 35.5%, and 34,3% of the DEGs in HK, spleen, and PerC B cells, respectively, were annotated into at least one KEGG pathway, while 39.3%, 46.7%, and 44.1% of the DEGs in HK, spleen, and PerC B cells, respectively, were linked to at least one over-represented GO term. At 3 wpi, DEGs from HK were enriched for a higher number of immune pathways compared to spleen (**Figures 5A, B**), while at 6 wpi, DEGs in spleen and PerC B cells were enriched for more immune pathways compared to HK B cells (**Figures 5C–E**). Additionally, HK B cells exhibited a reduced number of enriched immune-related pathways at 6 wpi compared to 3 wpi, while spleen B cells exhibited a higher number of enriched immune pathways at 6 wpi compared to 3 wpi. The KEGG analyses revealed enrichment of one or more canonical innate immune pathways, such as NOD-like, Toll-like, and RIG-I-like receptor signaling, as well as a cytosolic DNA-sensing pathway, in B cells obtained from all three sites and two time points, except spleen B cells at 3 wpi (**Figure 5B**). Moreover, pathways with known immunological functions, including extracellular matrix (ECM)-receptor interaction and phagosome pathways, were enriched only in spleen B cells at both time points (**Figures 5B, D**). Additionally, the p53 signaling pathway was enriched in B cells from the three sites and both time points except for HK B cells at 6 wpi (**Figure 5C**). The GO term analyses revealed enrichment of terms primarily linked with immune mechanisms (such as regulation of immune system process, immune response, defense response, immune system process, etc.) and cell divisions (including mitotic cell cycle process, nuclear division, cell cycle process, mitotic sister chromatin segregation, etc.) in B cells across the three sites suggesting actively dividing B cells (**Supplementary Figure 7**).

### 3.6 Antiviral and regulatory functions of Atlantic salmon B cells

**Figure 6:**
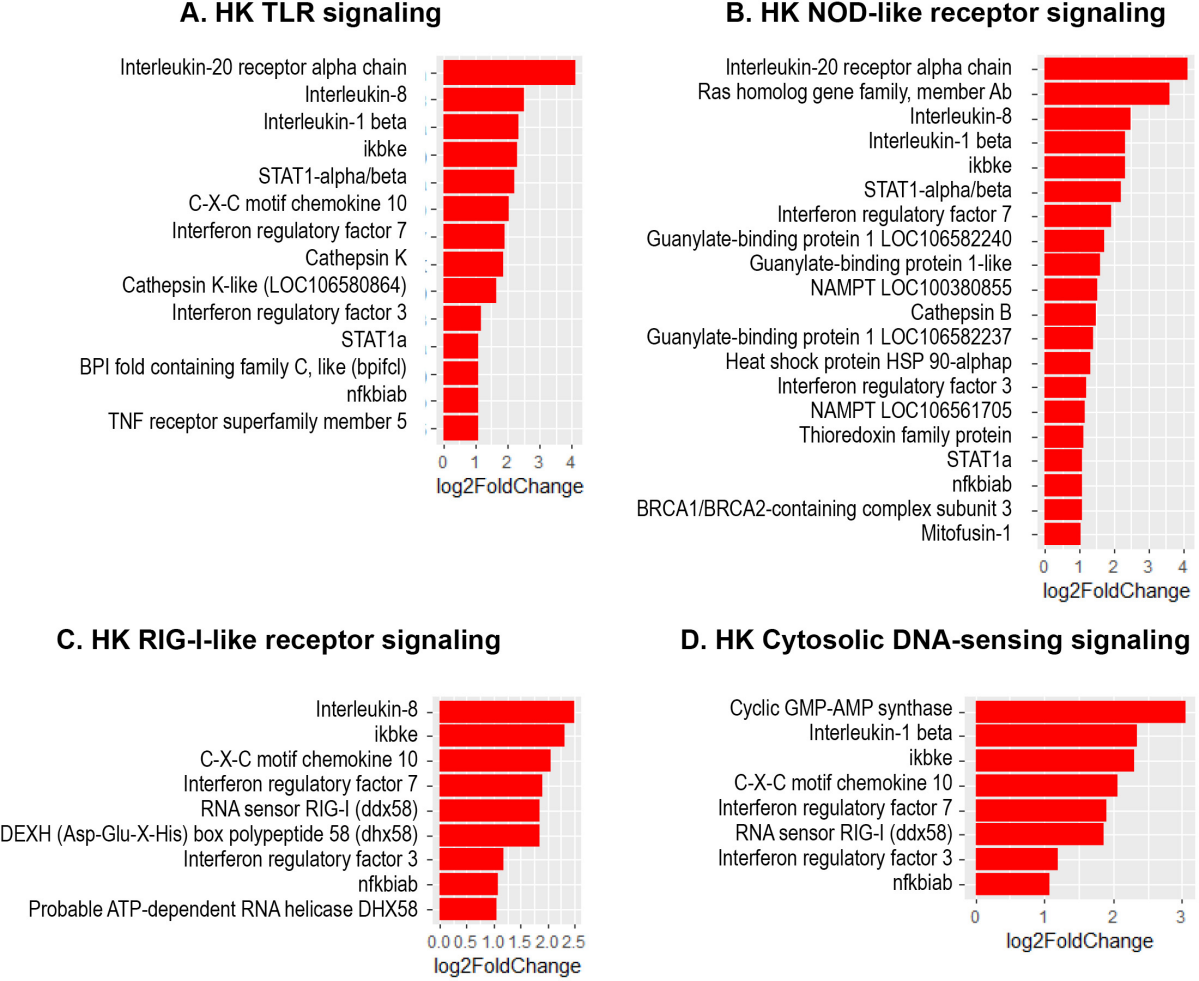
Genes representing enriched innate immune signaling pathways and their corresponding log2 fold change in HK at 3 wpi. **(A)** TLR-signaling pathway; **(B)** NOD-like receptor signaling; **(C)** RIG-I-like receptor; **(D)** Cytosolic DNA-sensing signaling.

**Figure 7:**
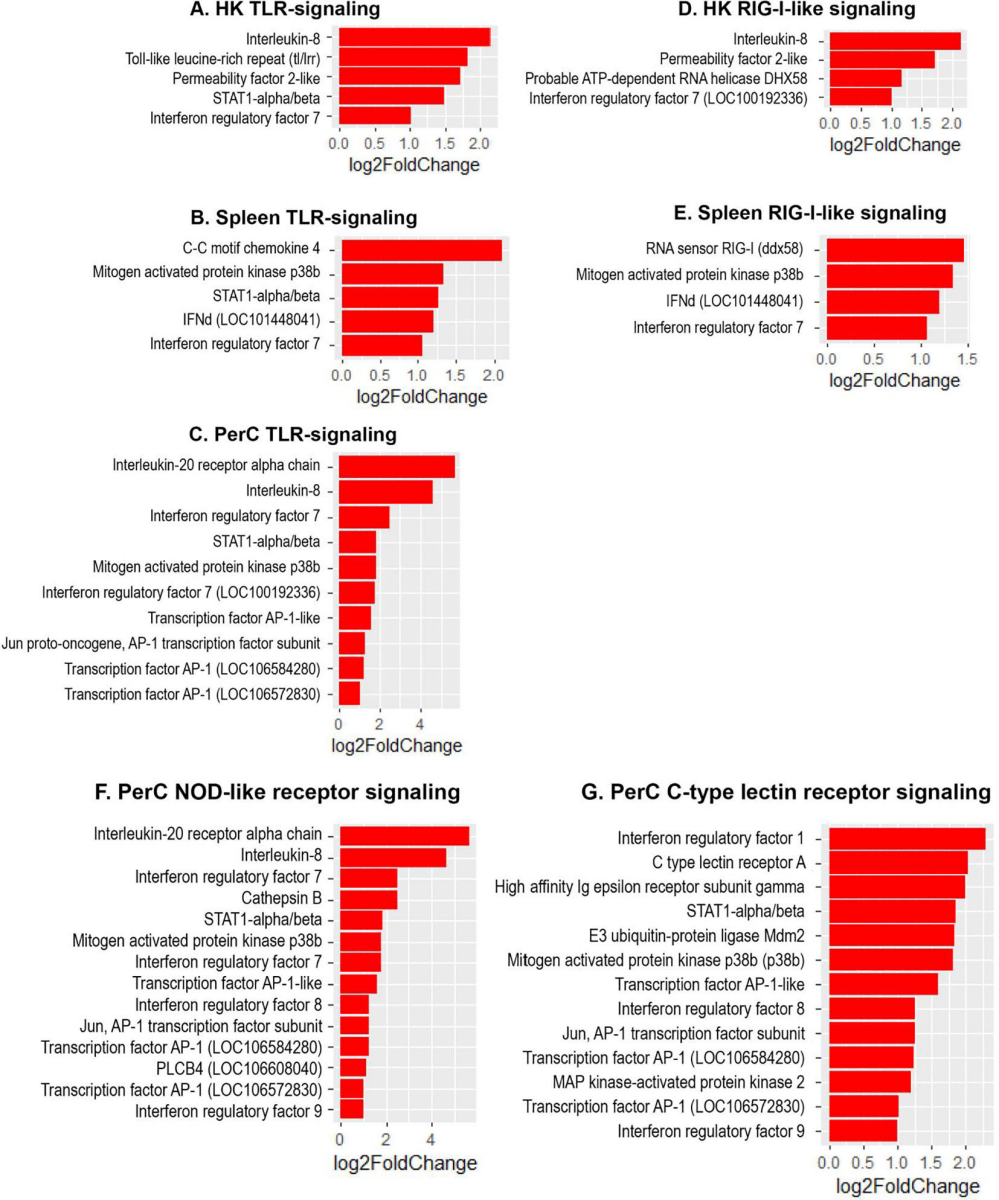
Genes representing enriched innate immune signaling pathways and their corresponding log2 fold change in HK, spleen and PerC at 6 wpi. **(A)** HK TLR-signaling; **(B)** Spleen TLR-signaling; **(C)** PerC TLR-signaling; **(D)** HK RIG-I-like receptor signaling; **(E)** Spleen RIG-I-like receptor signaling; **(F)** NOD-like receptor signaling; **(G)** C-type lectin receptor signaling.

**Figure 8:**
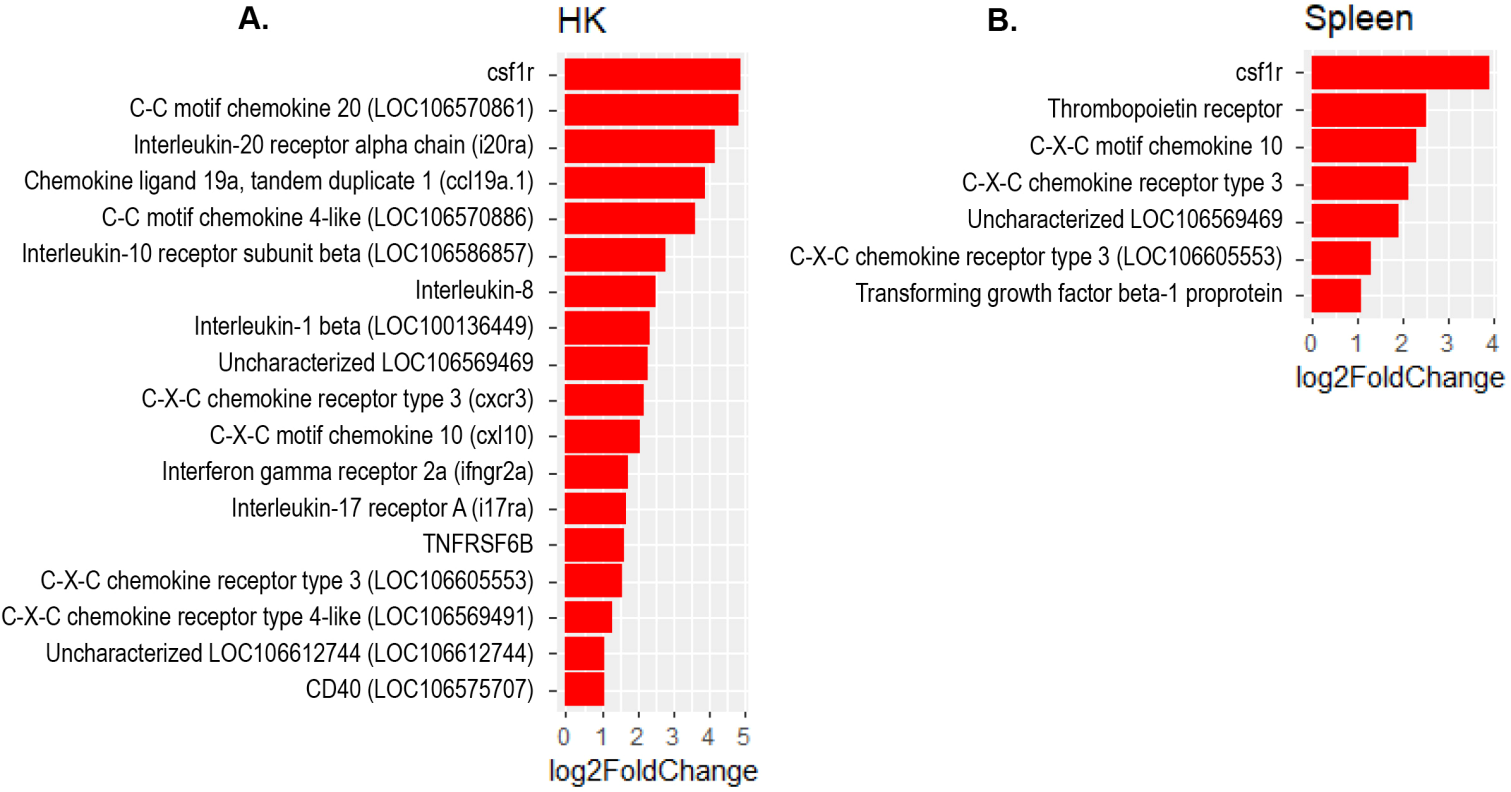
Genes representing the cytokine-cytokine interaction pathway and their corresponding log2 fold change in **(A)** HK and **(B)** Spleen B cells at 3 wpi.

**Figure 9:**
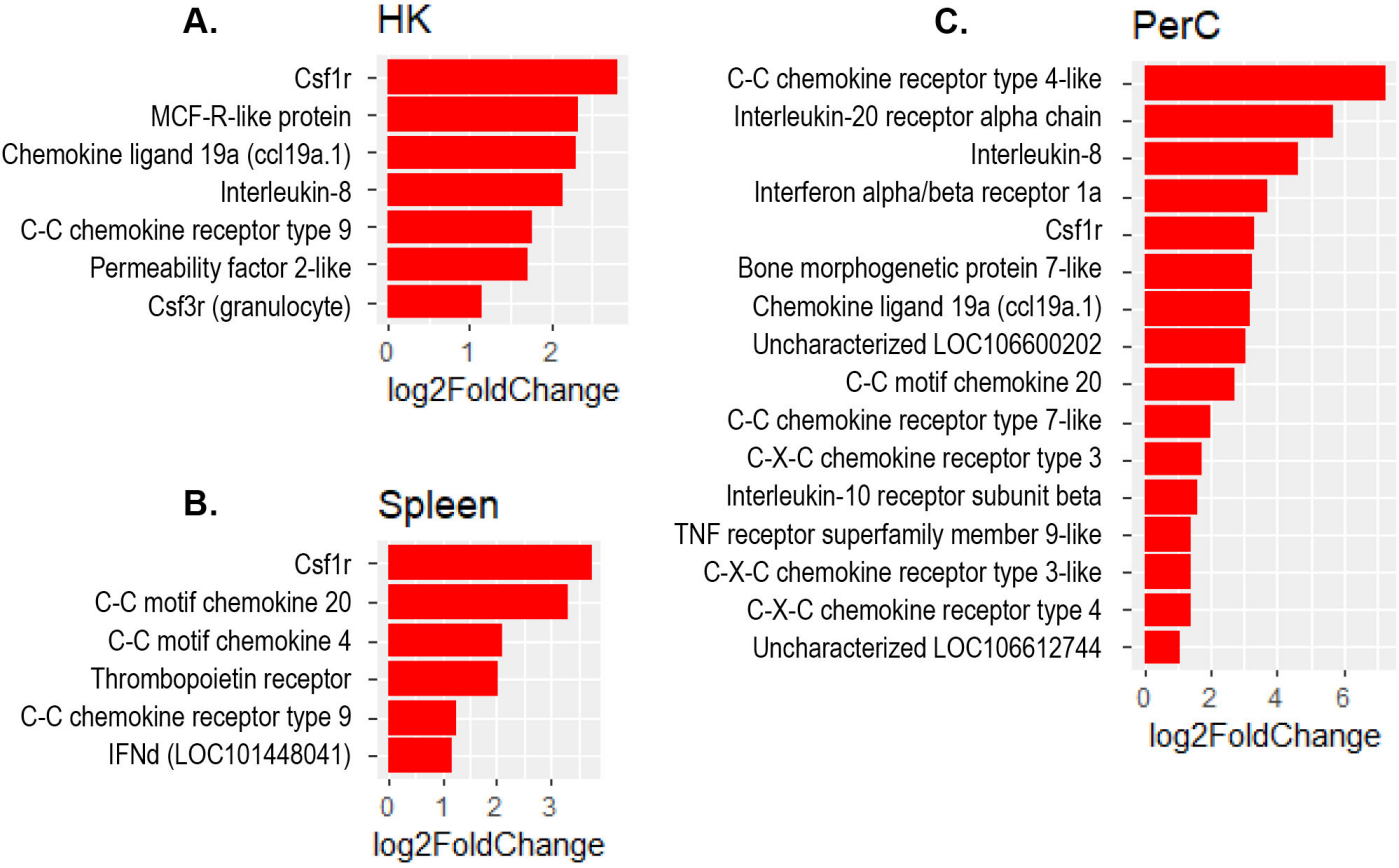
Genes representing the cytokine-cytokine interaction pathway and their corresponding log2 fold change in **(A)** HK, **(B)** Spleen and **(C)** PerC at 6 wpi.

The present study revealed a significant increase in the expression of several interferon regulatory factor (IRF) genes- namely *IRF1, IRF3, IRF7* and *IRF9* in Atlantic salmon B cells from the three sites, suggesting a SAV3-induced type I interferon (IFN) response. These IRF genes are shown under the different enriched innate immune pathways at 3 and 6 wpi (**Figures 6**, **7**). Additionally, the upregulation of *IFN*-alpha/beta receptor, along with upregulation of *STAT1*-alpha/beta and *IRF9*, emphasizes the effector function of Atlantic salmon B cells in response to type I IFNs (**Figures 6**, **7**). The *IRF8* gene also showed upregulation in PerC B cells at 6wpi (**Figures 7F, G**); however, whether it plays a role in regulating B cell activation, as reported in mammals (42), or is involved in other immune functions in fish B cells remains unknown. The enrichment of the MAPK signaling pathway in PerC B cells may indicate, among other things, antiviral mechanisms through modulating several cellular processes (**Figure 5E**, **Supplementary Figure 8**). Notably, among all Atlantic salmon type I IFN genes, only *IFNd* was upregulated in spleen B cells (**Figures 7B, E**). The study also identified enrichment of the cytokine-cytokine receptor interaction pathway with varying number of genes in B cells across the three sites (**Figures 8**, **9**). For instance, the number of enriched cytokine genes in HK B cells was more than twice that in the spleen at 3 wpi (**Figure 8**), while PerC B cells had more than twice as many enriched cytokine genes compared to HK or spleen B cells at 6 wpi (**Figures 9A–C**). Additionally, our analysis showed an upregulation of genes counteracting NF-κB signaling in HK B cells at 3 wpi, such as *nfkbia* (nuclear factor of kappa light polypeptide gene enhancer in B-cells inhibitor, alpha) and *ikbke* (inhibitor of NF-κB kinase subunit epsilon) (**Figures 6A, B**).

## 4 Discussion

Despite recent advances in the transcriptome profiling of teleost B cell subsets (20–22), many aspects of their functionalities remain poorly understood. The advances provide snapshots of events occurring at specific points in time, enabling a comprehensive profiling of fish B cell subsets from several immune tissues and identifying several potential population-specific markers, thus laying the foundation for future phenotypic and functional characterizations. Teleost B cells are known for their potent phagocytic and bactericidal capability that can prime naïve T cells, thereby linking the adaptive and innate immune systems (1). Although these capabilities have been recognized for over two decades, the innate signaling pathways leading to B cell activation and differentiation, the crosstalk between the different innate receptors and the BCR remain enigmatic.

### 4.1 Phenotypic characteristics of Atlantic salmon B cells

The IgM^+^IgD^+^ B cells represent the dominant subset in fish, especially in systemic immune tissues (17, 43, 44). Additionally, in most studied teleost species, naïve mature B cells co-express IgM and IgD on their surface (IgM^+^IgD^+^). Our results herein bring new insight into this important B cell subset and to the best of our knowledge this is the first time that Atlantic salmon B cells are characterized with an anti-IgD antibody. In the current study, IgM^+^IgD^+^ B cells from SAV3-infected Atlantic salmon exhibited phenotypic changes, including increased size and decreased surface expression of IgM and IgD, indicating their differentiation towards antibody-secreting cells (**Figures 1C, D**). This corresponds to our earlier findings in Atlantic salmon of a SAV3-induced increase in antibody-secreting cell responses (27, 28). Similar phenotypic changes have been observed in other fish species such as the trout (16, 24). Notably, we found a trend of increased frequency of IgM^+^IgD^-^ cells in SAV3-infected fish, particularly in the PerC, which could indicate a more terminally differentiated B cell population, as reported in rainbow trout (24). However, it remains unclear whether terminally differentiated B cells, also known as plasma cells, in fish retain surface IgM, as has been described in mature IgM-expressing plasma cells in mammals (45).

### 4.2 Transcriptionally and functionally distinct IgM^+^IgD^+^ B cells across the studied immune sites

This study showed differences in the number and type of SAV3-induced DEGs among B cells from HK, spleen and PerC. For instance, at 6 wpi, when B cells from all the three sites were analyzed, PerC B cells exhibited over twice as many DEGs compared to HK or spleen B cells, with only a limited overlap in the DEGs (**Figure 2A**). Similarly, a limited number of DEGs showed overlap in the most affected 100 DEGs ranked by LFC (**Figure 2C**). Together, these findings suggest the existence of distinct B cell subsets in each tissue, each exhibiting a unique gene expression pattern. However, it remains unclear whether the differences in DEGs are solely a result of varying B cell composition due to different maturation stages or if there are indeed phenotypically distinct B cell subsets with unique immune functions or perhaps a combination of both. To shed light on this, we explored the expression of PR domain zinc finger protein 1 (*BLIMP-1*; LOC106607555), also known as a plasma cell differentiation marker, which was upregulated in B cells from the three sites (**Figure 2D**). This suggests that the differences in the transcriptome profile of B cell at the three sites may not solely be due to differences in the stage of B cell maturation.

To date, a comprehensive study comparing transcriptional or phenotypic differences among B cell subsets from major lymphoid tissues in fish is lacking. A previous study in rainbow trout showed transcriptional heterogeneity in naïve IgM^+^ B cells across various tissues, indicating the existence of functionally distinct, tissue-specific B cells (19). Additionally, recent single-cell RNA-seq studies of B cells in rainbow trout and Atlantic salmon revealed several transcriptionally distinct clusters in peripheral blood, HK, and spleen, unveiling unprecedented diversity among fish B cells (20–22). However, it is yet to be determined whether these transcriptionally distinct B cell clusters correspond to phenotypically distinct subsets. Despite an increasing number of reports demonstrating the transcriptional diversity of B cells, there seems to be a tendency to overlook this, often considering them as a uniform population akin to mammalian B1 B cells. The current study is the first to compare the transcriptome responses of B cells from both systemic and peripheral immune sites in teleost species. The findings challenge the prevailing paradigm that views fish B cells as a uniform population, supporting previous claims of transcriptional complexity in fish B cells within and across different immune sites (19–22).

Despite variations in the transcriptome profile of Atlantic salmon B cells across the three sites investigated, functional enrichment analyses revealed a convergence in fundamental biological functions, such as innate-immune signaling, cell division, and regulation via cytokine-cytokine interaction pathways. This convergence is expected since genes responsible for maintaining basic biological activities tend to have a conserved expression pattern, resulting in enrichment of comparable functional pathways (46). Differences in enriched immune signaling pathways were also identified. For instance, only spleen B cells at both time points showed enriched ECM-receptor interaction and phagosome pathways (**Supplementary Figure 9**). In mice, a unique ECM niche has been described in spleen that supports a specialized population of marginal zone B cells that respond rapidly and are therefore crucial for the first line of defense (47). However, the interactions between fish B cells and ECM, as well as how this interaction regulates B cell functions, remain completely unknown. Although fish B cells are known for their phagocytic activity regardless of their location (48), the enrichment of the phagosome pathway only in spleen B cells in this study highlights the need to revisit our understanding of the process of phagocytosis and the sites of antigen presentation by fish B cells.

### 4.3 Transcriptome dynamics of IgM^+^IgD^+^ B cells

From 3 to 6 wpi, we found a notable decrease in the number of DEGs in HK and spleen B cells (**Figure 2A**). This finding suggests a reduction in the severity of pathological changes or infection, as the fish attempt to restore homeostasis. Previous studies have demonstrated a decline in viral RNA load in the heart of Atlantic salmon infected with SAV3 during the same period indicating a containment of viremia (27, 49). A correspondence between disease severity and virus load has been reported in a prior study of SAV3 infection in Atlantic salmon (50). Therefore, it is plausible to assume that the temporal change in the number of DEGs from 3 to 6 wpi could be linked to a decrease in infection. The overlap of DEGs between the two time points was only about 9% for HK and about 14% for spleen (**Figure 2E**). Nevertheless, assigning functional pathways to the DEGs that were maintained over the two time points was challenging, as many of them had only stable gene IDs and were incompatible with the annotation databases. In general, this was a limitation of the study, stemming from the incomplete annotation of the Atlantic salmon genome, which led to the exclusion of a considerable number of DEGs during functional enrichment analyses. Therefore, caution is necessary when interpreting these findings, as the exclusion of DEGs may preclude enrichment of relevant B cell signaling pathways.

### 4.4 IgM^+^IgD^+^ B cells exhibit innate immune capability

In the present study, we found enrichments of the major innate-immune signaling pathways, including TLR, RIG-I-like receptor, NOD-like receptor, C-type lectin receptor, and cytosolic DNA-sensing pathways in B cells from SAV3-infected Atlantic salmon (**Figures 5**, **7**). While several genes belonging to these pathways have been previously identified in various fish species at the tissue level or in macrophage-like cells, relatively little is known about their roles in specific leukocyte subsets (51–55). This study, therefore, presents a comprehensive report on the concurrent enrichment of various innate immune signaling pathways in fish B cells from systemic and peripheral immune sites. The concurrent enrichment of these major innate immune pathways supports prior findings that underscore the important innate immune functions of fish B cells (1, 48). Moreover, the findings provide valuable insights into the potential ability of fish B cells to integrate innate signals, thereby regulating the outcome of an immune response to infection. Despite the significant progress in characterizing PRR signaling pathways in fish B cells, challenges remain in understanding how PRRs are utilized by fish B cells to sense pathogens, leading to phagocytosis and downstream signaling (52, 56, 57). It is worth noting that, despite the high phagocytic capacity of fish B cells (48), the process of BCR-mediated phagocytosis remains elusive.

Interestingly, despite the observed phenotypic changes indicating the activation and differentiation of B cells, such as an increase in size and a decrease in surface IgM/IgD expression (**Figures 1C–E**), the BCR signaling pathway was not functionally enriched in the present study. This may be partly explained by the absence of Atlantic salmon IgM genes from the databases used for functional annotation. However, it should be noted that enriched pathways are typically represented by numerous DEGs, and the absence of a single DEG may not invariably determine whether a pathway is enriched. Furthermore, the investigation of downstream genes implicated in BCR signal transduction, including *syk, btk, akt, blyk, lyn, p38, CD79a/b, ZAP-70, RAS*, etc., exhibited no changes in expression in response to the SAV3 infection (**Supplementary Tables 2**-**6**). This could indicate BCR-independent B cell activation or that the activation involves phosphorylation of these downstream signaling molecules at the protein level without affecting their gene expression profile.

### 4.5 Immunoregulatory function of IgM^+^IgD^+^ B cells

The present study revealed an enrichment of the cytokine-cytokine receptor interaction pathway in B cells across the three sites. Notably, the specific genes showing enrichment differed among B cells obtained from these sites (**Figures 8**, **9**), suggesting existence of distinct B cells in these sites with different immunoregulatory function. Only a few studies have examined the expression of cytokine genes in fish B cells (58, 59), and functional studies have not kept pace. Based on the limited functional studies and gene homology searches in mammals, fish cytokines appear to have pleotropic functions (2, 59–61). However, it is uncertain to what extent these functions are conserved between fish and mammals. The enrichment of the cytokine-cytokine receptor interaction pathway with genes for both cytokine ligands and receptors, suggests a role for Atlantic salmon B cells as regulators and effectors of immune responses. Additionally, the upregulation of genes counteracting the action of NF-κB in HK B cells at 3 wpi, such as *nfkbia* and *ikbke*, suggests a potential role of B cells in restricting excessive inflammatory responses. In higher vertebrates, B cells are not only simply antibody ‘factories’ but also actively regulate immune responses by secreting diverse cytokines (62, 63). Teleost fish have phagocytic and professional antigen-presenting B cells (1, 2). Thus, it is expected that these cells can regulate the immune response by producing cytokines, as corroborated by the findings of the present study. Notably, the colony-stimulating factor 1 receptor (cfsr1) gene, a myeloid linage marker, was one of the DEGs with the highest fold-change in the cytokine-cytokine receptor signaling pathways in B cell from all three sites. While this could indicate gene expression plasticity in B cells, similar to mammalian B cell subsets that also express csf1r (64, 65), we cannot completely rule out the possibility of myeloid cell contamination in the sorted B cells.

### 4.6 Type I IFN responses in IgM^+^IgD^+^ B cells in fish

IRFs play a key role in regulating type I IFN responses (66, 67). In the current study, the upregulation of several IRF genes suggests their conserved role in mediating antiviral signals downstream of PRRs in fish B cells, which leads to the expression of type I IFN genes and IFN-stimulated genes. The upregulation of *IRF3* and *IRF7* at 3 wpi is consistent with earlier studies in teleost fish that identified them as true orthologues of their mammalian counterparts and key regulators of type I IFN gene expression (66, 68–73). Notably, the TLR and NOD-like receptor signaling pathways were enriched with two *IRF7* genes (LOC100192336 (*irf7A*) and LOC100329194 (*irf7B*)) in PerC B cells, with *IRF7B* showing a slightly higher expression than *IRF7A* (**Figures 7C, F**). A previous study in Atlantic salmon has also reported duplicate *IRF7* genes from diverse tissues that demonstrated varying effects on the type I IFN promoter (71). The biological implications of the existence of two *IRF7* genes require further studies. Additionally, only PerC B cells exhibited differential expression of *IRF1/8/9* within the NOD-like or C-type lectin receptor signaling pathway, indicating distinct regulatory mechanisms among B cells mediated by IRFs. Interestingly, only one of the six subtypes of Atlantic salmon type I IFN genes, *IFNd*, was upregulated in spleen B cells at 6 wpi. IFNd in Atlantic salmon has been previously reported to lack antiviral activity (74–76). In higher vertebrates, type I IFNs not only inhibit virus replication but also regulate B cell responses and thereby bridging the adaptive and innate immunity (77, 78). Whether IFNd has evolved to regulate the adaptive B cell response or has an undiscovered antiviral function in Atlantic salmon B cells is currently unclear.

In summary, the IgM^+^IgD^+^ B cells from HK, spleen, and PerC of SAV3-infected Atlantic salmon exhibited distinct transcriptional profiles, underscoring the complex nature of B cell subsets in fish. However, it remains to be determined whether this reflects specific differential stages of B cells at the studied sites or represent phenotypic variations giving rise to functional differences. The concurrent enrichment of various innate immune signaling pathways and the cytokine-cytokine receptor interaction pathway in B cells from all three sites indicate that their functions extend beyond the traditional role of antibody production. However, the phenotypic changes corresponding to B cell activation and differentiation occur without enrichment of the BCR signaling pathway. This calls for a deeper understanding of the molecular requirements for B cell activation and the underlying mechanisms governing B cell responses in fish. Gaining these insights could ultimately contribute to the development of efficacious vaccines capable of eliciting robust B cell responses.

## Data Availability

The datasets presented in this study can be found in online repositories. The names of the repository/repositories and accession number(s) can be found in the article/**Supplementary Material**.
