# Supplementary material for: Transcriptionally distinct B cell profiles in systemic immune tissues and peritoneal cavity of Atlantic salmon (Salmo salar) infected with salmonid alphavirus subtype 3
Source: Front Immunol. 2024 Dec 3;15:1504836. doi: 10.3389/fimmu.2024.1504836 (PMC11649679; doi:10.3389/fimmu.2024.1504836)
Supplement: Supplementary file 1 [file DataSheet1.docx]

Supplementary Material

**Supplementary Table 1.** IGHM gene sequences used for manual annotation

| **Accession No.** | **Description** |
| --- | --- |
| BT044763.1 | Salmo salar clone ssal-rgf-502-381 Ig mu chain C region membrane-bound form putative mRNA, complete cds |
| BT058539.1 | Salmo salar clone Contig1694 Ig mu chain C region membrane-bound form putative mRNA, complete cds |
| BT059185.1 | Salmo salar clone ssal-rgf-531-092 Ig mu chain C region membrane-bound form putative mRNA, complete cds |
| Y12456.1 | S.salar mRNA for IgM heavy chain 7.3 |
| BT058702.1 | Salmo salar clone Contig3502 Ig mu chain C region membrane-bound form putative mRNA, complete cds |
| S48652.1 | IgM CHA=immunoglobulin heavy chain constant region {clone g5.1} [Salmo salar=Atlantic salmon, mRNA, 1462 nt] |
| Y12457.1 | S.salar mRNA for IgM heavy chain 7.2 |
| GU129140.1 | Salmo salar IgH locus B genomic sequence |
| Y12392.1 | S.salar CHB(5.1) gene |
| Y12391.2 | S.salar CHA(6.3) gene |
| GU129139.1 | Salmo salar IgH locus A genomic sequence |
| AF269072.1 | Salmo salar immunoglobulin heavy chain variable region (IgH) mRNA, partial cds |
| AF273401.1 | Salmo salar clone 06-01 immunoglobulin heavy chain variable region (IgH) mRNA, partial cds |
| AF273400.1 | Salmo salar clone 01-02 immunoglobulin heavy chain variable region (IgH) mRNA, partial cds |
| AF273403.1 | Salmo salar clone 07-03 immunoglobulin heavy chain variable region (IgH) mRNA, partial cds |
| BT047556.2 | Salmo salar clone ssal-rgb2-633-364 Ig heavy chain V-I region HG3 precursor putative mRNA, complete cds |
| AF273402.1 | Salmo salar clone 06-14 immunoglobulin heavy chain variable region (IgH) mRNA, partial cds |
| AF269073.1 | Salmo salar clone 03-15 immunoglobulin heavy chain variable region (IgH) mRNA, partial cds |
| AF269074.1 | Salmo salar clone 09-05 immunoglobulin heavy chain variable region (IgH) mRNA, partial cds |
| AF273407.1 | Salmo salar clone 08-09 immunoglobulin heavy chain variable region (IgH) mRNA, partial cds |
| AF273434.1 | Salmo salar clone 02-13 immunoglobulin heavy chain variable region (IgH) mRNA, partial cds |
| AF273415.1 | Salmo salar clone 05-12 immunoglobulin heavy chain variable region (IgH) mRNA, partial cds |
| AF273404.1 | Salmo salar clone 01-05 immunoglobulin heavy chain variable region (IgH) mRNA, partial cds |
| AF273421.1 | Salmo salar clone 08-05 immunoglobulin heavy chain variable region (IgH) mRNA, partial cds |
| AF273406.1 | Salmo salar clone 04-01 immunoglobulin heavy chain variable region (IgH) mRNA, partial cds |
| AF273408.1 | Salmo salar clone 09-09 immunoglobulin heavy chain variable region (IgH) mRNA, partial cds |
| AF269083.1 | Salmo salar clone 09-06 immunoglobulin heavy chain variable region (IgH) mRNA, partial cds |
| BT049245.1 | Salmo salar clone ssal-evf-534-265 Ig heavy chain V-I region HG3 precursor putative mRNA, complete cds |
| AF273409.1Length: 440 | Salmo salar clone 10-01 immunoglobulin heavy chain variable region (IgH) mRNA, partial cds |

**Supplementary figures**


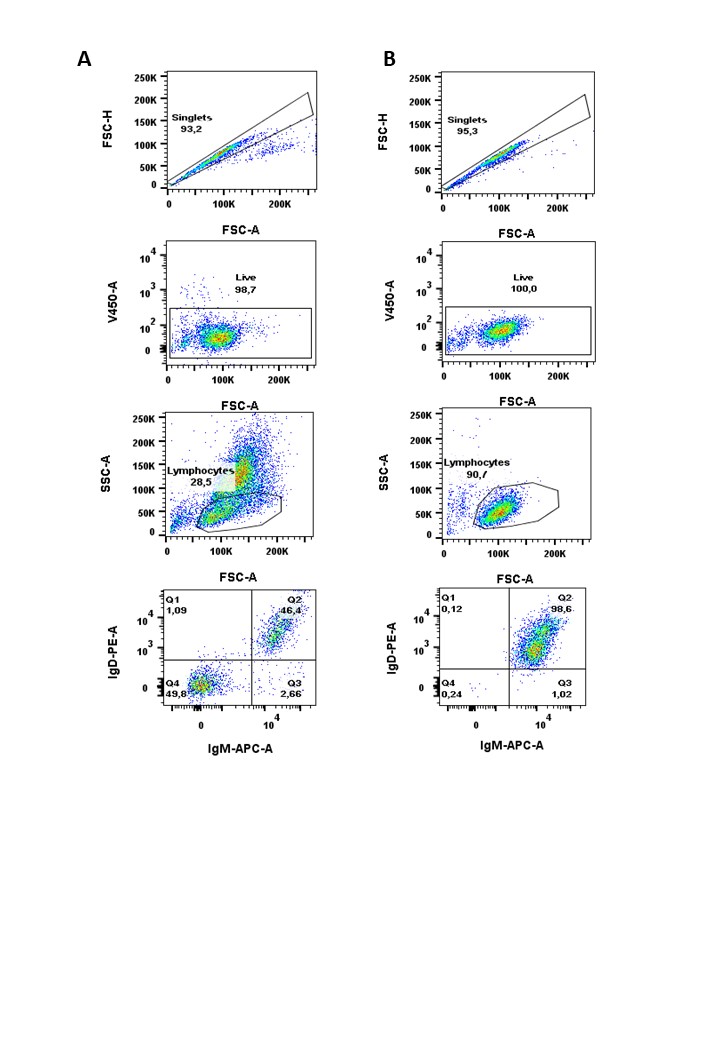


Supplementary Figure 1. Gating strategy and analysis of IgM^+^IgD^+^ cell purity. Leukocytes were stained as described in the materials and methods section. (A) Doublets (FSC-A Vs FSC-H) and dead cells were excluded, followed by gating for lymphocyte. IgM^+^IgD^+^, IgM^+^IgD^-^, and IgM^+^IgD^+^ cells were sorted. (B) The purity of the sorted IgM^+^IgD^+^ cells was assessed using FACS by sorting aliquots of cells into the sorting buffer.


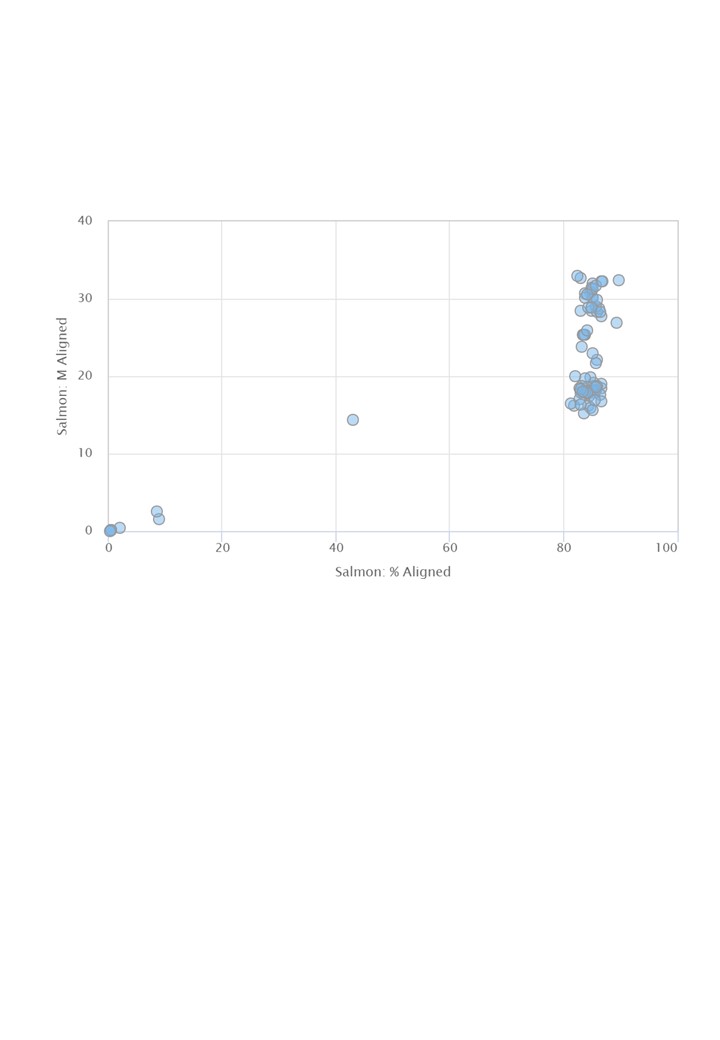


**Supplementary Figure 2.** Mapping rate of sequences. Sequences were mapped using the Salmon program, and only samples with mapping rate above 80% were included in further analyses.


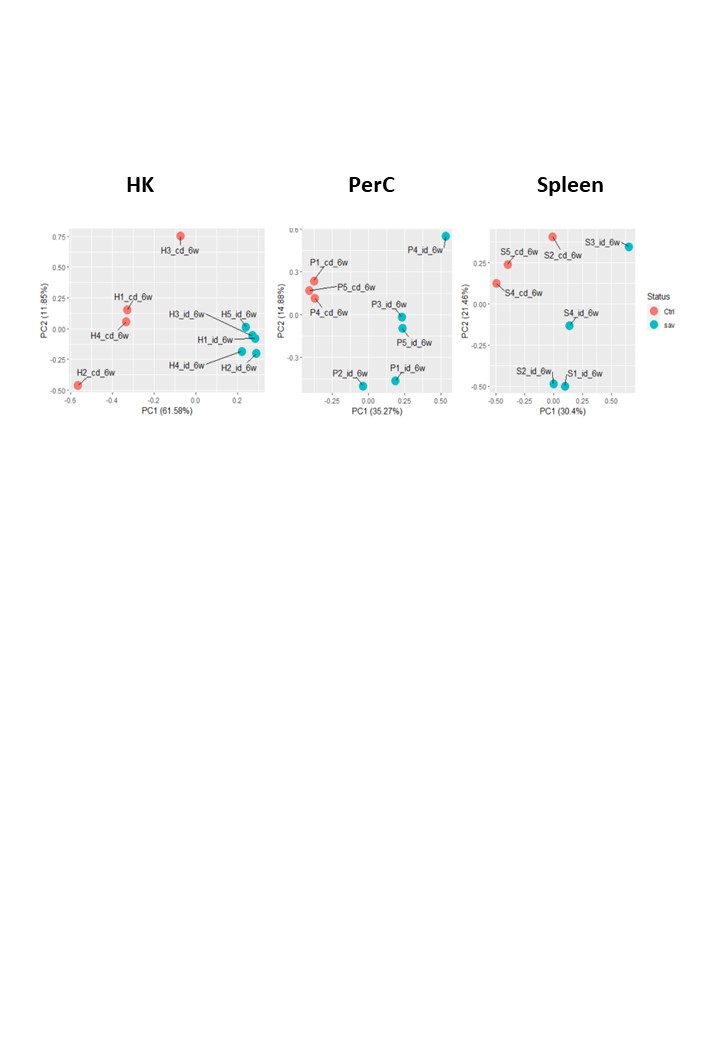


**Supplementary Figure 3.** Principal component analysis of head kidney (HK), peritoneal cavity (PerC) and spleen B cell samples at 6 wpi. Red dots represent B cell samples from control fish, while samples from SAV3-infected Atlantic salmon are represented by blue dots.


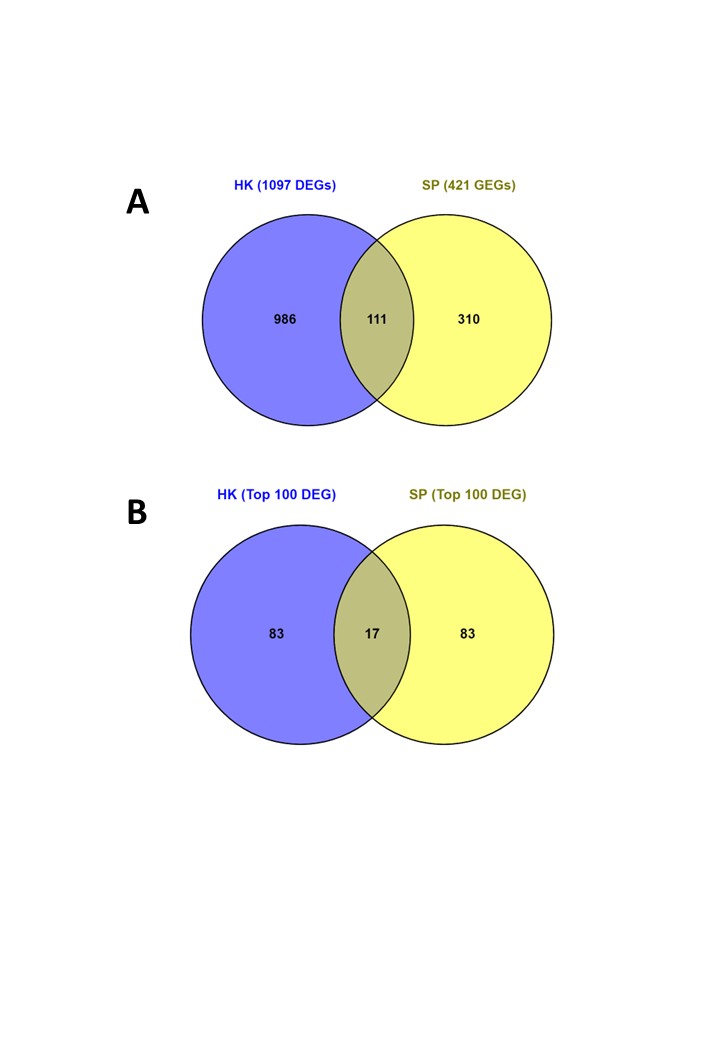


**Supplementary Figure 4.** Venn diagram showing the total number of differentially expressed genes (DEGs) and DEGs shared between HK and spleen B cells at 3 wpi. (A) Total number of DEGs, and (B) the top 100 DEGs ranked by log2 Fold Change (LFC).

**
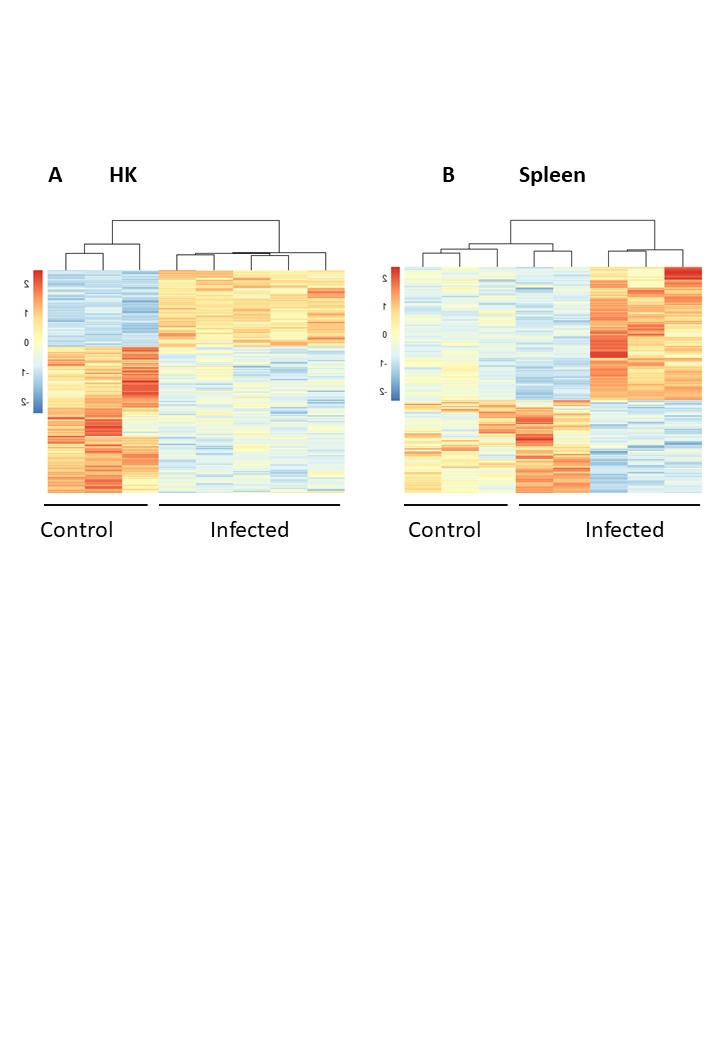
**

**Supplementary Figure 5.** Heat map and hierarchical clustering of the DEGs in (A) HK and (B) spleen B cells from control and infected fish at 3 wpi. The side bar from 2 to -2 indicates Z-score, where the red color represents upregulated genes, while the blue color represents downregulated genes.

**A**


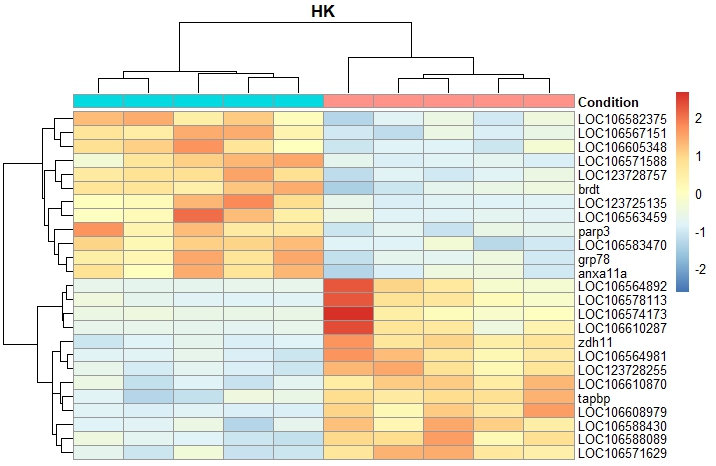


**B**


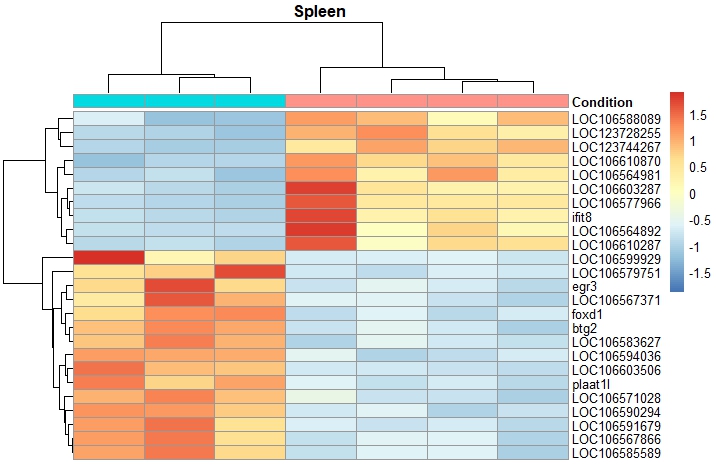


**C**


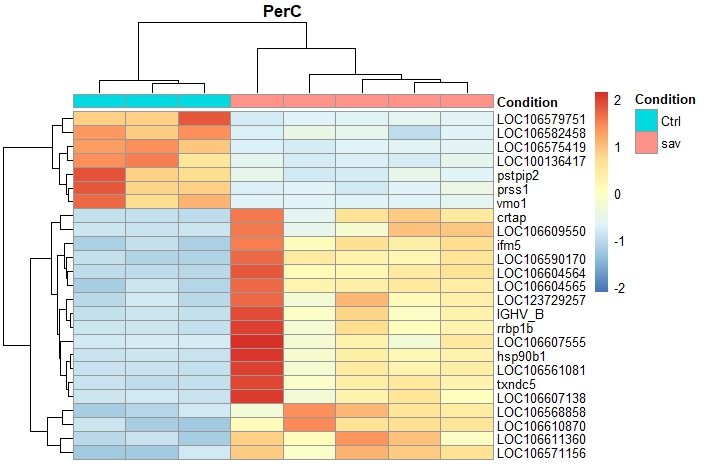


**Supplementary Figure 6.** Heat map and hierarchical clustering of the top 25 DEGs with their gene names in (A) HK, (B) spleen and (C) PerC B cells from control and infected fish at 6 wpi. The side bars indicate Z-score, where the red color represents upregulated genes, while the blue color represents downregulated genes.


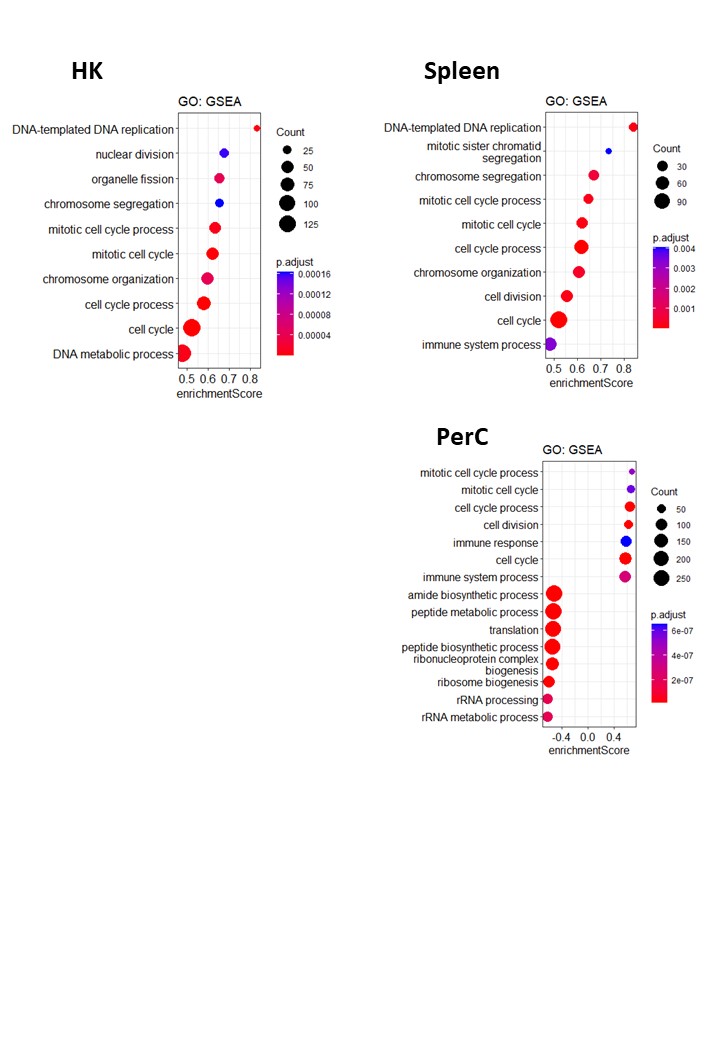


**Supplementary Figure 7.** GO analyses showing overrepresented terms related to immune mechanisms and cell division at 6 wpi


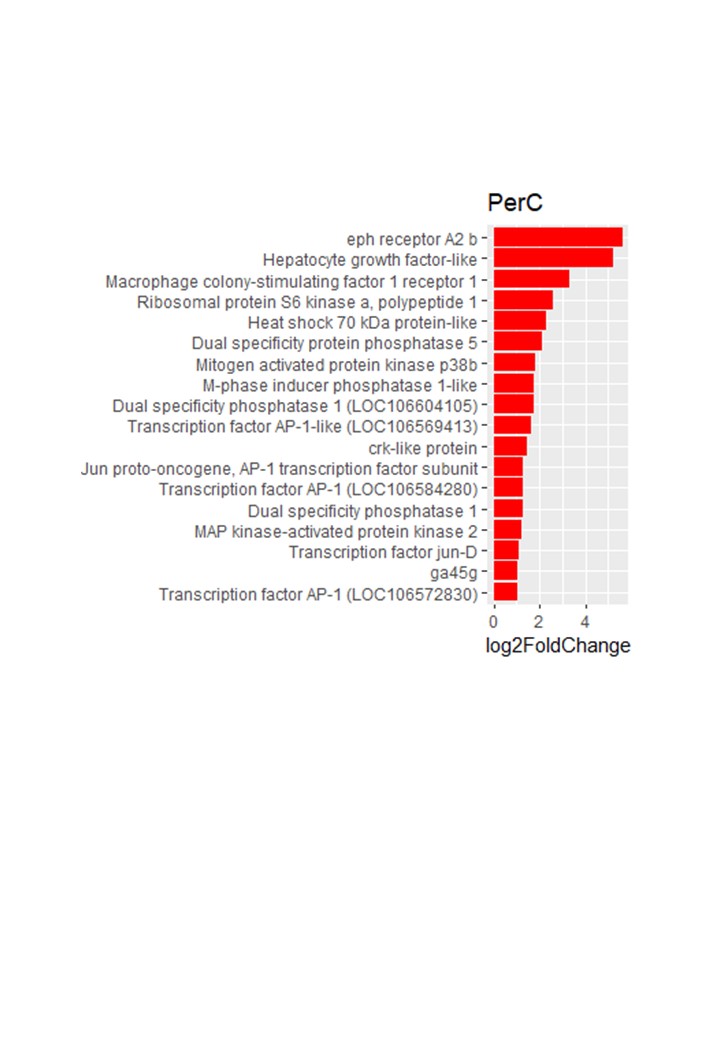


**Supplementary Figure 8.** Enriched MAPK signaling pathway in PerC B cells at 6 wpi.


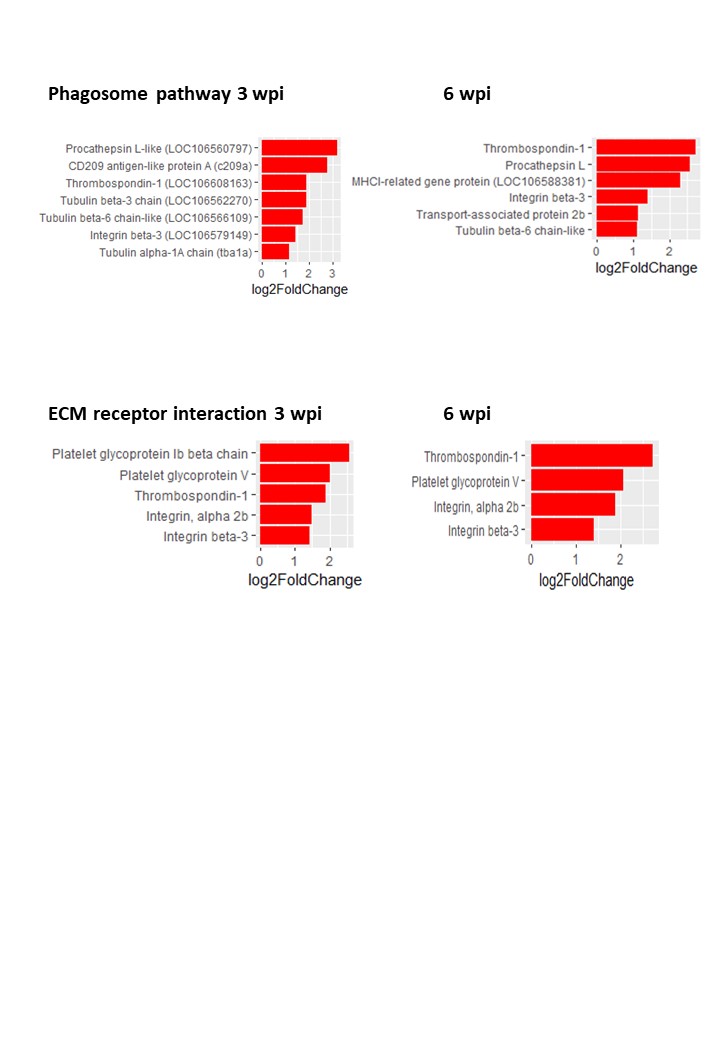


**Supplementary figure 9**. Enriched phagosome and ECM receptor interaction pathways in spleen B cells at 3 and 6 wpi
